# Micro/nanofabrication of heat management materials for energy-efficient building facades

**DOI:** 10.1038/s41378-024-00744-y

**Published:** 2024-08-26

**Authors:** Guanya Wang, Keunhyuk Ryu, Zhaogang Dong, Yuwei Hu, Yujie Ke, ZhiLi Dong, Yi Long

**Affiliations:** 1 Department of Electronic Engineering, The Chinese University of Hong Kong, Shatin, New Territories, 999077 Hong Kong SAR, China; 2 School of Materials Science and Engineering, Nanyang Technological University, Singapore, 639798 Singapore; 3 Institute of Materials Research and Engineering (IMRE), Agency for Science, Technology and Research (A*STAR), 2 Fusionopolis Way, Innovis #08-03, Singapore, 138634 Singapore; 4 School of Interdisciplinary Studies, Lingnan University, Tuen Mun, New Territories, 999077 Hong Kong SAR, China

**Keywords:** Nanoscale materials, Optical materials and structures

## Abstract

Advanced building facades, which include windows, walls, and roofs, hold great promise for reducing building energy consumption. In recent decades, the management of heat transfer via electromagnetic radiation between buildings and outdoor environments has emerged as a critical research field aimed at regulating solar irradiation and thermal emission properties. Rapid advancements have led to the widespread utilization of advanced micro/nanofabrication techniques. This review provides the first comprehensive summary of fabrication methods for heat management materials with potential applications in energy-efficient building facades, with a particular emphasis on recent developments in fabrication processing and material property design. These methods include coating, vapor deposition, nanolithography, printing, etching, and electrospinning. Furthermore, we present our perspectives regarding their advantages and disadvantages and our opinions on the opportunities and challenges in this field. This review is expected to expedite future research by providing information on the selection, design, improvement, and development of relevant fabrication techniques for advanced materials with energy-efficient heat management capabilities.

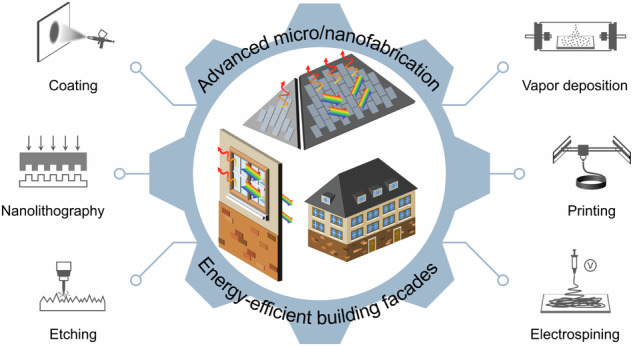

## Introduction

Despite the rapid advancements in clean energy technologies, the global energy supply continues to be heavily reliant on traditional fossil fuels^1^, resulting in a variety of environmental and human health issues^2,3^. Improving energy efficiency and reducing carbon emissions are imperative for ensuring the sustainability of human society. Currently, buildings comprise a significant portion of global energy consumption, accounting for ~32% of final energy usage, ~51% of electricity consumption, and ~33% of carbon emissions^4–6^. The building energy sector surpasses the industry and transportation sectors, representing ~40% of total social energy usage^7–9^. Within the building sector, building services, including heating, ventilation, and air conditioning, contribute to ~50% of energy consumption^7^. Developing energy-efficient building facades is a promising solution, particularly for reducing the energy consumption necessary to maintain indoor thermal comfort. Conventional energy-efficient building facades primarily focus on improving thermal resistance to reduce conductive heat transfer between indoor and outdoor environments. Recently, significant research interest has been focused on managing heat transfer through electromagnetic radiation, specifically by controlling the solar irradiation^10,11^ and thermal emission^12,13^ properties of building facades. In terms of thermal emissions, buildings are best designed to dissipate heat to cold outer space through radiation in the wavelength range of 8–13 μm, which is known as the atmospheric window^14^.

**Fig. 1:**
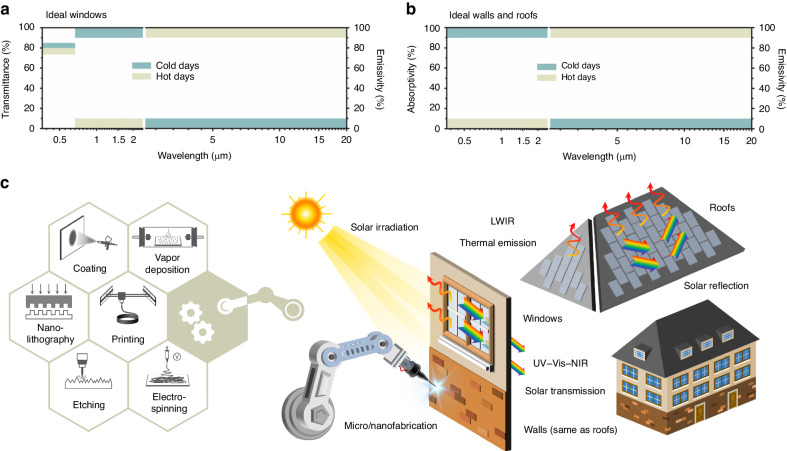
Concepts associated with this review. **a** Ideal spectral properties for energy-efficient windows^27^. **b** Ideal spectral properties for energy-efficient walls and roofs^27^. Reproduced with permission from the American Chemical Society, copyright 2022. **c** Schematic diagram of the review outline

Windows, walls, and roofs are essential components of building facades, each with distinct optical and thermal property requirements. Windows that transmit sunlight and exchange heat serve as crucial interfaces for buildings, yet they often exhibit lower energy efficiencies than other building components^15,16^. Extensive efforts have been devoted to solar transmittance management in windows using smart (also called dynamic or stimuli-responsive) chromogenic materials that can modulate indoor sunlight^17–20^, mainly in the visible (Vis) and near-infrared (NIR) bands. Through on-demand control of indoor solar irradiation, smart windows effectively reduce energy usage in building air conditioning systems. It was recently stated that compared to plain glass windows, thermochromic windows can reduce heating and cooling energy demand by ~5.0–84.7%, according to the research progress reported from 2009 to 2019^21^. Another review involving simulations suggested that commercial electrochromic windows can reduce the total amount of energy delivered by 6–40%, depending on the region^11^. Luminous transmission (*T*_lum_) and solar transmission modulation (Δ*T*_sol_) are two important indicators for evaluating the performance of these windows. Δ*T*_sol_ represents the difference in solar transmission between transparent and opaque states^22^. In general, energy-efficient smart windows should have a high Δ*T*_sol_ and a moderate *T*_lum_, as the former parameter determines the regulation of solar irradiation, while the latter parameter maintains appropriate indoor luminance levels for visual comfort^23,24^. Recently, it was demonstrated that dynamic control of thermal emission in the longwave infrared (LWIR) range is crucial for promoting the energy efficiency of smart windows^25–29^. Therefore, an ideal energy-efficient smart window is expected to exhibit high transmittance in the NIR band and low emission in the LWIR band during cold days and to exhibit low transmittance in the NIR band and high emission in the LWIR band during hot days (Fig. 1a)^27^.

Energy-efficient walls and roofs differ from windows due to their intrinsic nontransparency^30,31^. In addition to thermal emission, energy-efficient walls and roofs primarily require the material property of solar absorption rather than transmittance^32^. Ideal energy-saving wall and roof are expected to exhibit high absorption in the ultraviolet (UV)−Vis−NIR band and low emission in the LWIR band during cold days and to exhibit low absorption in the UV−Vis−NIR band and high emission in the LWIR band during hot days (Fig. 1b)^27^. Although dynamic modulation shows great potential, research in this area is much less extensive than that concerning static materials with subambient radiative cooling capabilities, which aims to minimize solar absorption and maximize thermal emission. This disparity may be attributed to the challenging cost and complexity associated with dynamic building facades and to the vast potential of radiative cooling applications beyond building facades^33–37^. Static radiative cooling materials alone are effective in reducing building energy consumption than traditional building facade materials. In a simulation study, a 5000 m^2^ office building model was built with 60% of its roof area (984 m^2^) serving as the variable to assess the energy savings from daytime radiative cooling materials^38^. The results revealed that compared to roofs without cooling materials, building models with cooling materials show cooling load reductions of 10% in Miami, 17–36% in Las Vegas, 61–84% in Los Angeles, more than 90% in San Francisco, and 26–63% in Chicago during the cooling season (June to September).

Advanced micro/nanofabrication techniques greatly contribute to heat management materials for energy-efficient building facades, especially in terms of material property design. There are several reviews summarizing material aspects, such as materials for smart windows^10,23,39–42^, radiative cooling structures^12,43,44^, and dynamic thermal emissivity control^45,46^. However, there is a lack of systematic reviews discussing fabrication methods. In this work, we present the first focused review on fabrication methods, offering a comprehensive summary of recent developments in micro/nanofabrication techniques for heat management materials that have the potential to be applied to energy-saving windows, walls and roofs (Fig. 1c). Considering the abovementioned research, we focus more on solar transmittance modulation than on thermal emission modulation for discussion related to windows and more on radiative cooling than heating or switchable emission for discussion related to walls and roofs. Additionally, we include some promising radiative cooling materials that we believe to be potentially applicable to energy-efficient windows, walls and roofs. The fabrication methods include coating, vapor deposition, nanolithography, printing, etching, and electrospinning. We detail the mechanisms by which spectral properties of materials are tailored in these fabrication methods and provide our opinions on the advantages and disadvantages of different fabrication techniques. Finally, we discuss the opportunities and challenges that lie ahead. This review is anticipated to accelerate future research by offering perspectives on the selection, design, improvement, and development of related processing methods for the enhancement of heat management material performance.

## Coating

Coating is a process in which functional materials cover substrate surfaces. Coating is considered a facile and effective method for producing interfacial functional films. This method is particularly advantageous due to its easy accessibility, large scale, low cost, rapid processability, and high suitability for diverse materials. In this section, we discuss typical coating techniques utilized in the manufacturing of functional coatings with solar modulation and/or radiative cooling capabilities, including spray coating, dip coating, spin coating, and roll-to-roll processing.

### Spray coating

Spray coating, dip coating, and spin coating are solution-based coating methods that involve applying liquid solutions or suspensions onto surfaces. Spray coating involves specialized guns or nozzles that disperse solutions in the form of tiny droplets that are sprayed, resulting in the rapid formation of large films on substrates^47^. Notably, this technique is superior to other coating techniques in terms of the fabricated coating area (mass production) because of the high freedom of movement of spray guns or nozzles.

**Fig. 2:**
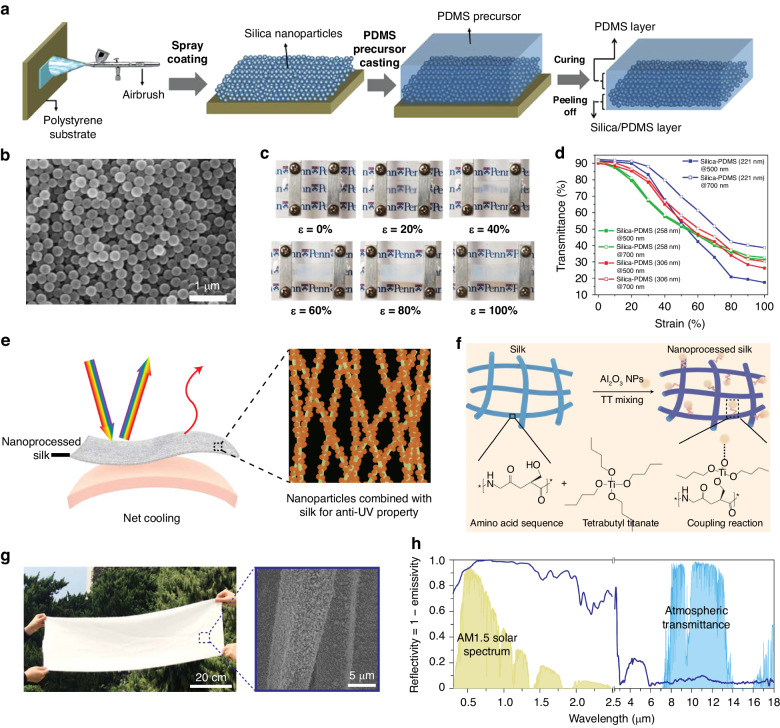
Spray coating and dip coating fabrication. **a** Schematic diagram of the fabrication process for a smart window based on SiO_2_ NP/PDMS composite films^48^. **b** SEM image of the quasiamorphous SiO_2_ arrays^48^. **c** Optical images of the composite film under different strains^48^. **d** Relationship between the transmittance of the films made of SiO_2_ NPs with different diameters at 500 nm and 700 nm and the applied strain^48^. Reproduced with permission from John Wiley and Sons, copyright 2015. **e** Schematic diagram of the net cooling of nanoprocessed silks achieved by attaching Al_2_O_3_ NPs with UV-reflection properties^76^. **f** Schematic diagram of TT facilitating the bonding of Al_2_O_3_ NPs and silk fibers through the coupling reaction^76^. **g** Optical image and SEM image of nanoprocessed silk^76^. **h** Reflectivity spectrum of nanoprocessed silk in 0.3–18 μm^76^. Reproduced with permission from Springer Nature, copyright 2021

The homogeneity of functional coatings is a key factor in the proper functioning of smart windows. Spray coating allows for the relatively good distribution of optical nanomaterials, including nanoparticles (NPs)^48–50^, nanowires (NWs)^51–53^, and nanotubes (NTs)^54^. For example, Ge et al. demonstrated a mechanically modulated smart window based on elastomeric poly(dimethyl siloxane) (PDMS) films embedded with quasiamorphous arrays of silica (SiO_2_) NPs^48^. The fabrication process is shown in Fig. 2a. The quasiamorphous arrays were prepared by spraying SiO_2_ NPs four times at a distance of 5 cm and a movement speed of ~5 cm/s. The scanning electron microscopy (SEM) image in Fig. 2b shows that the SiO_2_ NPs were assembled into long-range disordered and short-range ordered structures. The SiO_2_/PDMS composite films could switch between high transparency and structural opaqueness under mechanical force modulation (Fig. 2c). Both the transmittance and the structural color were influenced by the diameter of the SiO_2_ NPs (Fig. 2d). Yu et al. fabricated photothermal coatings by spray assembling gold (Au) NP colloids on substrates^49^. Deposition by spraying at a pressure of 30 pounds per square inch (psi) for 20 s could achieve ~80% of the saturation deposition achieved by dipping for 2 h. The light-to-thermal conversion of thermoplasmonic AuNPs effectively facilitated the thermochromic switch of the poly(N-isopropylacrylamide) (PNIPAm) hydrogel to modulate solar transmittance. In addition, spray coating was determined to be feasible for producing random networks of one-dimensional (1D) nanomaterials with high aspect ratios^55^. Liu et al. developed interconnected silver (Ag) NW networks with desired optical and electrical properties by spraying ethanol and AgNW solutions onto preheated hydrophobic Teflon plates^51^. The networks were then transferred onto PDMS to create transparent and stretchable electrodes for elastomeric electrochromic windows.

Walls and roofs constitute most of the exterior area of a building. As an efficient preparation method already widely used in the construction industry, spray coating is an ideal choice for painting large-area radiative cooling materials^56–60^. For example, Chen et al. synthesized an inorganic phosphoric acid-based geopolymer paint and subsequently fabricated coatings with a thickness of ~50 μm by spraying the precursor at a pressure of 2 MPa and a distance of ~300 mm for a duration of 3 s^57^. The solar reflectivity and thermal emissivity of the coatings were estimated to be 90% and 95%, respectively. The high emissivity was also maintained over a wide range of incident angles from 0 to 75°. The spectral properties were proven to be stable in different severe environments, including high-temperature, mechanical abrasion-based, and proton irradiation-based environments. Therefore, spray coating should drive the commercialization of radiative cooling materials and provide a sustainable solution for environmentally friendly buildings.

### Dip coating

Dip coating relies on a process that draws out immersing substrates from functional material solutions, offering an easily accessible feature that requires minimal precision equipment. In practical applications, brush painting is considered a relatively simplified method of dip coating.

This method has been extensively employed to fabricate chromogenic coatings for windows, including vanadium dioxide (VO_2_)-based thermochromic coatings^61–69^ and tungsten oxide (WO_3_)-based electrochromic coatings^70–73^. For example, Cao et al. used dip coating and freeze drying to produce nanoporous VO_2_ thin films on fused SiO_2_, where the film thickness increased with increasing withdrawal speed, leading to a higher Δ*T*_sol_ and a lower *T*_lum_^61^. Ke et al. further improved the method using prepatterned substrates to produce nanostructured VO_2_ films. The researchers reported that the affinity between vanadium precursors and substrates is important for precisely controlling nanoscale structures, and an oxygen-plasma treatment to make the substrate hydrophilic is essential for the successful coating of water-based precursors regardless of their viscosity^63^. Deepa et al. reported that WO_3_ films obtained through dip coating exhibited better electrochromic performance and cycling and chemical stabilities than those obtained through spin coating^70^. Wang et al. produced mesoporous WO_3_ films for smart windows with integrated optical modulation and energy storage capabilities, reaching a high optical modulation of 75.6% at 633 nm^71^. In addition to WO_3_-based electrochromic devices, Salles et al. developed an optimized dip coating method to synthesize titanium carbide (Ti_3_C_2_T_x_) films, which acted as transparent conductive electrodes and electrochromic active materials^74^. Flake size, solution concentration, and repeated dip times were considered the main factors for the optimization of the Ti_3_C_2_T_x_ preparation scheme.

The building energy efficiency could be enhanced by painting cooling materials or by covering cooling films on walls and roofs. Silkworm materials inherently possess high thermal emissions^75^ and have the advantages of scalability and low prices, making them suitable for large-area production. Nevertheless, solar absorption needs to be addressed for commercial silks when implemented in subambient radiative cooling applications. Zhu et al. designed a molecular bonding strategy based on coupling reagent-assisted dip coating to adhere aluminum oxide (Al_2_O_3_) NPs onto silks to enhance UV reflectivity (Fig. 2e)^76^. After the dipping and drying processes, the coupling reagent tetrabutyl titanate (TT) was connected to the Al_2_O_3_ NPs via hydrogen bonds and to the silks via covalent bonds, as shown in Fig. 2f. Al_2_O_3_ NPs were uniformly distributed on silk (Fig. 2g). The application of Al_2_O_3_ NPs promoted reflectivity across the full solar spectrum without affecting the high emissivity of the silks (Fig. 2h). As a result, a temperature ~3.5 °C lower than the ambient temperature under direct sunlight was observed on the nanoprocessed silk surface. Similarly, Li et al. imparted cooling capability and hydrophobicity to silk fabrics by sequentially immersing and drying them in Al_2_O_3_ and octadecyltrichlorosilane solutions^77^.

### Spin coating

Spin coating is a reliable method for producing films with adjustable thicknesses. This technique depends on the centrifugal force generated by high-speed rotation to evenly spread solutions over substrate surfaces. By tuning the rotation speed and/or the number of spin coatings, the film thickness can be effectively controlled. The size of spin-coated products is usually on the subinch scale. The following discussion is a summary of the applications of spin coating in energy-saving windows with single- and dual-band optical characteristics.

Several studies have demonstrated the production of thickness-controllable VO_2_ films using well-designed spin coating processes for realizing the desired thermochromic performance^78–82^. Kim et al. employed a two-step spin coating approach for preparing VO_2_ films^80^. The first step involved low-speed rotation (500 rpm) for 30 s, followed by the second step of high-speed rotation (3000–5000 rpm) for an additional 30 s to control the film thickness. After intense pulsed light sintering, the optimal VO_2_ films exhibited a Δ*T*_sol_ of 11.2% and a *T*_lum_ of over 46.3%. Rashid et al. repeated the spin coating process five times using the VO_2_ precursor solution to generate thicker films than those prepared by repeating the process three times, leading to light absorption enhancement^81^. This technique could also be used to produce chromogenic coatings of other materials with controllable thicknesses. Yu et al. reported a coating solution containing organometallic [(C_2_H_5_)_2_NH_2_]_2_NiCl_4_, cesium tungsten bronze (Cs_x_WO_3_), and antimony tin oxide (ATO) for thermochromic smart windows^83^. An optimized Vis transmittance of ~70% and NIR shielding of ~90% were attained by setting the spin speed to 1500 rpm at a fixed solution concentration. Cao et al. chose tantalum-doped titanium dioxide (Ta−TiO_2_) nanocrystals as the electrochromic material to fabricate a smart window featuring three operation modes (bright, cool, and dark)^84^. The dark mode corresponding to the Vis blocking function was attained by five repeated spin coating processes. In addition, static radiative cooling contributes to the energy efficiency of windows, but it requires high transmittance in the solar band, which mostly agrees with the requirements of cooling coatings for solar cells^85^. Zhou et al. reported that PDMS features negligible solar absorption and reduced reflection in the atmospheric window because methyl groups and vinyl-terminated cross-linkers are bonded on the chains of alternating silicon and oxygen atoms^86^. The scholars optimized the cooling performance of PDMS films for windows by controlling the rotation speed during the spin coating process. A solar absorptivity of 0.4% and an LWIR emissivity of 90% could be attained in the 80-μm-thick PDMS films.

**Fig. 3:**
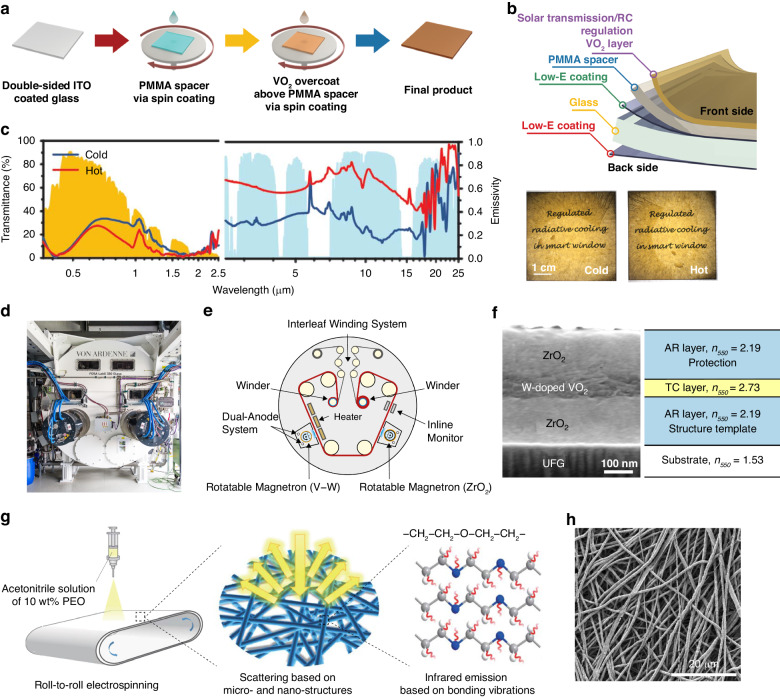
Spin coating and roll-to-roll processing fabrication. **a** Schematic diagram of the preparation process for a thermochromic window with passive radiative cooling regulation ^25^. **b** Structural diagram and optical images of the smart window^25^. **c** Vis−NIR transmittance and LWIR emissivity spectra of the smart window in cold and hot states^25^. Reproduced with permission from the American Association for the Advancement of Science, copyright 2021. **d** Optical image of the large-scale roll-to-roll device for manufacturing ZrO_2_/W–VO_2_/ZrO_2_ thermochromic coatings^95^. **e** Schematic diagram of the large-scale roll-to-roll device^95^. **f** Cross-sectional SEM image and functional diagram of the ZrO_2_/W–VO_2_/ZrO_2_ coating^95^. Reproduced with permission from Elsevier, copyright 2022. **g** Schematic diagram of the fabrication of hierarchical POE NF films and the mechanism of net cooling^103^. **h** SEM image of the random stacked NFs of the polymer film^103^. Reproduced with permission from Springer Nature, copyright 2020

As previously mentioned, additional thermal emission management for dual-band control properties could further improve the energy-saving performance of smart windows. Wang et al. introduced an innovative design for thermochromic windows with passive radiative cooling regulation by sequentially spin coating poly(methylmethacrylate) (PMMA) and VO_2_ on glass plates coated with double-sided indium tin oxide (ITO), as shown in Fig. 3a^25^. The stacked multilayer structure of VO_2_/PMMA/ITO acted as a Fabry−Perot resonator (Fig. 3b). Experiments and simulations revealed that there was a critical relationship between the cooling regulation capability and the thickness of the PMMA spacer. A maximum difference in the emissivity of 40% between the hot and cold states could be achieved by adjusting the spin speed during the PMMA layer preparation. Figure 3c shows that the smart window maintained solar modulation while automatically regulating radiative cooling performance. Lin et al. designed a hydrogel-based smart window with thermal regulation in which different AgNW layers were fabricated via spin coating on PNIPAm to provide heat insulation in cold states^87^. Compared to single-layer coatings, an improvement of 46% in thermal reflectivity was observed when double-layer AgNW coatings were applied. When the temperature was above the phase transition temperature of PNIPAm, the water/PNIPAm connected structures were destroyed because of weakened hydrogen bonds, and then a layer of water was formed to increase the thermal emissivity and suppress the thermal reflectivity of the AgNW coatings. Recently, Wang et al. reported a novel dual-band regulation smart window with solar transmittance and thermal emission management capabilities by integrating kirigami-structured PDMS with AgNW-coated PNIPAm^88^.

### Roll-to-roll processing

Roll-to-roll processing is a continuous fabrication strategy that involves feeding, processing, and receiving films through multiple rolls. This manufacturing technique offers mass production and economic advantages, making it highly valuable for practical applications in the construction industry. Yet the cost will increase if other expensive techniques are involved.

Various smart windows have been reported based on roll-to-roll processing, including thermochromic windows^89,90^, electrochromic windows^91,92^, and liquid crystal (LC) windows^93,94^. Roll-to-roll processing is often integrated with other fabrication techniques, such as deposition, lithography, printing, and electrospinning, thereby holding great commercialization potential. Rezek et al. successfully transferred the manufacturing of thermochromic coatings of zirconium dioxide (ZrO_2_)/W–VO_2_/ZrO_2_ from a laboratory-scale device to a large-scale roll-to-roll deposition device (Fig. 3d–f), which marked a promising step toward industrial-scale production^95^. Compared to the samples prepared in the laboratory, the coatings produced by the scalable device had similar hysteresis loops and the same transition temperatures. Thus, it was verified that the thermochromic properties were almost the same. Deng et al. reported the successful fabrication of graphene/NW/plastic transparent electrodes using roll-to-roll processing integrated with chemical vapor deposition (CVD), hot lamination, and electrochemical delamination techniques^96^. These electrodes exhibited a high transmittance of ~94% at 550 nm with a low sheet resistance of ~8 Ω/sq and a long cycle life reaching 10000 cycles in poly(3,4-ethylenedioxythiophene) (PEDOT) electrochromic devices. The properties were benefited from the structural characters that metal NWs were covered by a large-area continuous graphene layer and were partially embedded into the plastic, where the NW junctions underwent fusion and flattening during the hot lamination process. Lin et al. prepared Ag nanofiber (NF)-based transparent electrodes for electrochromic windows via a roll-to-roll method combined with blow spinning and UV irradiation^97^. The precursor solution was injected onto a continuous rolling track to form fiber networks. Then, the networks were irradiated with four side-by-side UV lamps for Ag^3+^ reduction. This approach enabled the large-scale production of AgNF electrodes with comparable properties to those prepared by high-temperature sintering. Roll-to-roll sputtered multilayer electrodes for electrochromic window applications have also been reported^98–100^.

This method has wide application potential for cooling films for walls and roofs. By using the roll-to-roll deposition method, Zhu et al. successfully prepared large-scale prototypes of cellulose nanocrystals and ethylcellulose bilayer films that were structurally colored with subambient surface temperatures to promote their application in buildings^101^. Zhai et al. demonstrated the meter-scale production of a daytime cooling hybrid metamaterial made of a transparent polymer with randomly distributed internal SiO_2_ microspheres (MSs)^102^. The scholars claimed that this high-throughput and low-cost manufacturing of metamaterials is crucial for promoting radiative cooling as an achievable energy technology. Additionally, a roll-to-roll electrospinning technique has been developed to manufacture large-area polymer NF films for thermal emission^103–105^. Taking hierarchical polyethylene oxide (PEO)-based films as an example, this polymer consisted of only C–C, C–O, and C–H bonds that contributed to a favorable absorption band within the atmospheric window, and the resulting NFs had diameters comparable to the solar wavelength (Fig. 3g)^103^. Then, POE NFs were formed into randomly stacked structures (Fig. 3h) by the collection of rolling drums during the roll-to-roll process. As a result, the fabricated POE films exhibited a high reflectivity of more than 96.3% in the solar spectrum and a high emissivity of 78% in the atmospheric window. This approach could greatly advance the large-scale fabrication of polymer NF-based cooling materials.

## Vapor deposition

Vapor deposition utilizes chemical or physical processes to transport materials in the form of gas or vapor onto substrate surfaces, resulting in the formation of thin films. While vapor deposition generally requires more expensive equipment and complex operational procedures than the coating techniques described above, it offers advantages in terms of film quality control, such as thickness, purity, and interfacial bonding. Moreover, this approach is well suited for substrates with prefabricated sophisticated structures down to the nanoscale. This method commonly requires a long processing time and results in films with compact sizes, typically on the inch scale.

### Chemical vapor deposition

CVD operates through chemical reactions between precursor molecules and introduced gases in chambers. The process requires proper heating and a vacuum environment to induce chemical reactions and thin-film deposition.

CVD is frequently employed to fabricate chromogenic thin films for smart windows. For example, VO_2_ thin films with high quality and diverse morphologies can be prepared by controlling chemical reactions in the gas phase^106^. Several types of CVD, including atmospheric-pressure CVD (APCVD)^107–109^, low-pressure CVD (LPCVD)^110,111^, and plasma-enhanced CVD (PECVD)^112^, have been proven useful for modifying the thermochromic performance of VO_2_. Specifically, Warwick et al. produced VO_2_ with a reduced transition temperature through electric field-assisted APCVD, where the application of an electric field played a crucial role in modifying the microstructure^107^. To reduce the deposition temperature, Guo et al. employed LPCVD to produce pure monoclinic VO_2_ thin films using vanadium(III) acetylacetonate as the precursor^110^. The deposition time and annealing temperature were used to determine the thermochromic properties of the VO_2_ films. In addition, Matamura et al. presented high-quality VO_2_ films synthesized via mist CVD using a water-based precursor solution^113^. The good thermochromic properties were attributed to the fact that the water solution inhibited V^3+^ and V^5+^ contamination during the deposition process.

**Fig. 4:**
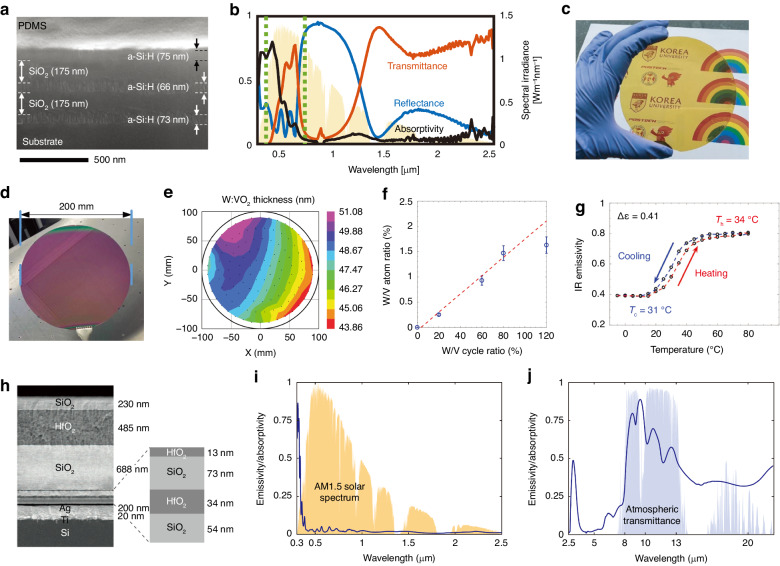
Vapor deposition fabrication. **a** Cross-sectional SEM image of the transparent cooler consisting of PDMS, a-Si:H, and SiO_2_^114^. **b** Transmittance, reflectivity, and absorptivity spectra of the transparent cooler in 0.4–2.5 μm^114^. **c** Optical image of the transparent cooler^114^. Reproduced with permission from John Wiley and Sons, copyright 2021. **d** Optical image of the W:VO_2_ film prepared with a W/V cycle ratio of 0.8^117^. **e** Thickness map of the W:VO_2_ film with a W/V atom ratio of 1.43%^117^. **f** Relationship between the W/V atom ratio and the W/V cycle ratio^117^. **g** Full infrared emissivity hysteresis curve of the W:VO_2_ reflector with a W/V ratio of 1.63%^117^. Reproduced with permission from John Wiley and Sons, copyright 2022. **h** Cross-sectional SEM image of the photonic cooler based on the multilayer structure of HfO_2_ and SiO_2_^130^. **i** Absorptivity spectrum of the photonic cooler in the UV−Vis−NIR region^130^. **j** Emissivity spectrum of the photonic cooler in the LWIR region^130^. Reproduced with permission from Springer Nature, copyright 2014

Multilayer photonic structures produced through multistep CVD could be used for daytime cooling windows, walls, and roofs. For instance, Kim et al. developed a transparent NIR reflector with a five-layered structure composed of alternating hydrogenated amorphous silicon (a-Si:H) and SiO_2_ (Fig. 4a)^114^. The reflector was integrated with a PDMS-coated glass substrate to form a transparent radiative cooler. The cooler effectively blocked a significant portion of NIR light from sunlight while transmitting substantial Vis light (Fig. 4b). Because of the low transmittance in the blue light region, the cooler was transparent and yellow (Fig. 4c). Conversely, Ma et al. utilized PECVD to create a seven-layered structure consisting of SiO_2_ and silicon nitride (Si_3_N_4_)^115^. The multilayer design not only contributed a high sunlight reflectivity of ~97% due to the optical impedance mismatch but also exhibited a high emissivity of ~75% in the atmospheric window through complementary phonon resonances.

### Atomic layer deposition

Atomic layer deposition (ALD) is a special type of CVD with ultrahigh precision that enables deposition with single-atomic-layer precision. The atomic precision allows for extremely uniform and reproducible films with excellent coverage, making them highly attractive in the field of nanodevices. This method is acknowledged as a highly expensive and time-consuming fabrication. The application of ALD in meter-scale production, such as for building facades, is challenging, while research on this technique at the laboratory stage will be valuable for understanding material properties. Li et al. initially grew thin films of amorphous vanadium oxide (V_x_O_y_) on glass, followed by a transformation to vanadium pentoxide (V_2_O_5_) and to high-purity VO_2_ through a series of postannealing processes^116^. The produced 50-nm-thick VO_2_ films exhibited a transition behavior at 65.6 °C, which was accompanied by a transmittance change of 43%. Notably, Sun et al. successfully prepared W-doped VO_2_ (W:VO_2_) films at the wafer scale using ALD^117^. As shown in Fig. 4d, e, a W:VO_2_ film was fabricated on a 200-mm wafer with a thickness gradient of less than 10 nm. A good linear relationship was observed between the W/V atom ratio and the W/V cycle ratio during the deposition process (Fig. 4f), which could effectively and precisely regulate the transition temperature of the final products. The phase transition behavior at room temperature was achieved at a W/V atomic ratio of 1.63% (Fig. 4g). The researchers further prepared a W:VO_2_ metasurface for adaptive radiative cooling and demonstrated an emissivity difference of more than 40% between hot and cold states.

### Physical vapor deposition

Physical vapor deposition (PVD) involves the physical transformation of materials from a solid to a gaseous state using tools such as heat, lasers, and electromagnetic fields. PVD is known to produce high-purity materials, as it does not involve the introduction of impurities from chemical reactions.

PVD has found extensive application in the field of smart windows, with numerous studies focusing on VO_2_ thin films. This technique offers a convenient and effective method for controlling the stoichiometry, crystallinity, microstructure, and performance of VO_2_^118^. Various PVD methods, including sputtering^119–122^, evaporation^123–125^, and pulsed laser deposition,^126–128^ have been proven to be effective. For example, Vu et al. utilized high-power impulse magnetron sputtering to prepare composite films comprising an amorphous V_2_O_5_ matrix embedded with VO_2_ nanorods^122^. The VO_2_ nanorods were produced through a guided growth method involving short-duration vanadium seeding and delayed oxygen injection during the sputtering process. These films exhibited a reduced transition temperature of 56.6 °C and good stability, with an estimated service life approaching 33 years. Recently, Bhupathi et al. reported a Fabry−Perot resonator based on evaporated zinc selenide (ZnSe) and sputtered VO_2_^129^. VO_2_ was characterized by porous structures because of oblique angle deposition, which allowed the Fabry−Perot resonator to have a higher *T*_lum_ and a much lower emissivity in cold states than those of dense VO_2_.

PVD is also promising for developing next-generation energy-saving walls and roofs. Similar to CVD, multilayer photonic structures can be produced through multistep PVD. In 2014, for the first time, Raman et al. achieved subambient cooling under direct sunlight using a multilayer photonic radiative cooler^130^. The cooler consisted of a bottom-up stack of Ti and Ag and of seven interleaved layers of hafnium dioxide (HfO_2_) and SiO_2_ (Fig. 4h), which were deposited by electron beam evaporation. In the interleaved layers, the bottom four layers with thicknesses of less than 100 nm played a role in the optimization of solar reflection, while the thick top three layers mainly contributed to thermal emission. Consequently, the cooler was endowed with a selective emissivity in the atmospheric window (Fig. 4I, j). In addition, a simplified strategy was proposed to achieve daytime cooling, which involved the use of PVD-based solar reflectors integrated with high-emissivity polymers^131–133^. For instance, Haechler et al. proposed a selective emitter containing a sandwich structure of chromium (Cr)/Ag/Cr produced via thermal evaporation, where a thin layer of Ag functioned as a sunlight reflector to minimize solar absorption, and Cr layers were formed to improve adhesion and prevent Ag oxidation^132^.

## Nanolithography

Nanolithography is an effective method for precisely fabricating two-dimensional (2D), two-and-a-half-dimensional (2.5D), and three-dimensional (3D) structures at the micro/nanoscale and is widely used in integrated circuit fabrication. Currently, nanolithography broadly refers to the creation of patterns with sample sizes ranging from a few nanometers to tens of millimeters^134^. Micro/nanostructures play a crucial role in designing material photonic properties^135,136^. In this section, we discuss several nanolithography techniques that are commonly employed in managing solar irradiation and thermal emission, including colloidal lithography, electron beam lithography (EBL), photolithography, and nanoimprint lithography (NIL). Because these techniques all involve multiple intricate processes, they are considered to be time intensive.

### Colloidal lithography

Colloidal lithography features masks made of self-assembling colloidal particles^137^. The method has been proven to be scalable and to produce samples several square meters in size^138^, which are extremely challenging when using other lithography methods, such as EBL (as discussed in the following subsection). However, defects are generally considered inevitable in these scalable samples^139^. Mask preparation is a highly important factor in determining cost. The raw materials of size-standard polystyrene (PS) nanospheres (NSs) are commonly sold for several hundred dollars per gram on the market.

This technique has been successfully applied to demonstrate a range of VO_2_ films with different 2D patterns^63,140–144^, which contributes to the controllable thermochromic performance in smart windows. The masks are critical for assisting in the fabrication of ordered arrays during etching, deposition, and surface modification processes. Size-standard NSs, which are typically made of polymers or SiO_2_, are the most commonly used colloidal particles due to their high tunability^139^. Combined with posttreatment processing, diverse microstructures with adjustable properties can be obtained. For example, Ke et al. utilized PS NSs as a monolayer colloidal template to create periodic VO_2_ nanoarrays with tunable morphologies, including NPs, nanodomes, and nanonets^63^. In the experiment, the vanadium precursor was deposited onto substrates masked by colloidal templates. The morphologies of the final products were tuned by changing the precursor viscosity and/or modifying the gaps among the PS NSs via oxygen-plasma treatment. The ability to efficiently control size and morphology could provide an opportunity to investigate localized surface plasmon resonance.

**Fig. 5:**
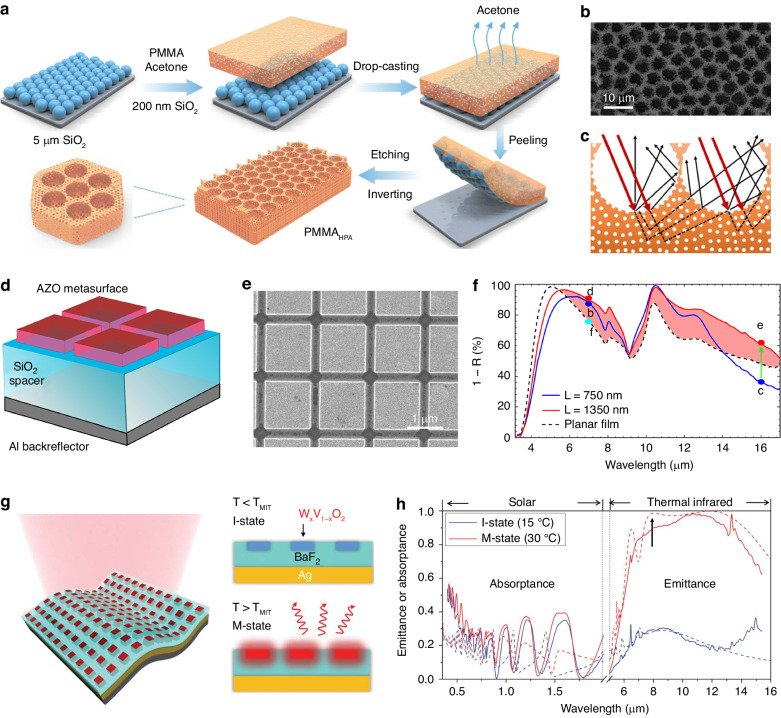
Nanolithography fabrication. **a** Schematic diagram of the fabrication process for PMMA films with hierarchically porous arrays^145^. **b** SEM image of the hierarchically porous arrays^145^. **c** Schematic diagram of the total scattering efficiency enhancement mechanism induced by micro/nanostructures^145^. Reproduced with permission from Springer Nature, copyright 2021. **d** Structural diagram of the AZO metasurface-based optical solar reflector^152^. **e** SEM image of the AZO metasurface^152^. **f** Emissivity spectra of AZO metasurfaces with different feature sizes and planar films^152^. Reproduced with permission from the American Chemical Society, copyright 2018. **g** Structural diagram and operation mechanism of the temperature-adaptive radiative cooler^156^. **h** Emissivity spectra of the cooler in the solar and thermal infrared regions at different states^156^. Reproduced with permission from the American Association for the Advancement of Science, copyright 2021

Regarding walls and roofs, the scalability and versatility of colloidal lithography have led to the exploration of materials with LWIR emission properties. Wang et al. introduced a method using SiO_2_-based masks to prepare PMMA films with hierarchical structures featuring micropore arrays and random nanopores, as illustrated in Fig. 5a, b^145^. The formation of micro/nanostructures was ascribed to the etching of colloidal SiO_2_ MSs and NSs, which enhanced the solar scattering and LWIR emission (Fig. 5c). Inspired by the broadband reflection splitting effect observed in the scales of four Nymphalid butterflies, Liu et al. developed a bilevel platinum (Pt) resonator with a 2D close-packed disk array pattern^146^. The upper and lower Pt disks were prepared with different periods and diameters by varying the size of the colloidal PS NSs and the etching time, which yielded specific optical properties, including tunable reflection under visible light, low specular reflectivity in 0.8–1.6 μm, selective radiation in 5–8 μm, and absorption at 10.6 μm.

### Electron beam lithography

EBL offers a direct method for writing patterns onto substrates, enabling the creation of intricate and precise patterns with high resolution down to the nanoscale^147^. This fine patterning of materials or structures at the micro/nanolevel is helpful for modulating optical properties^148^. Yuce et al. revealed that the phase transition temperature of VO_2_ decreased to nearly room temperature after the films were engraved by electron beams^149^. Similar to ALD, the application of EBL, together with photolithography (as discussed in the following subsections), in the construction field could face great challenges because of its low throughput and high cost. However, laboratory-based investigations could be informative for further understanding the fundamental properties of smart materials.

Metasurfaces featuring nanoarrays have emerged as an effective solution for selective thermal emitters. Recent studies have shown that EBL could be adapted and utilized for radiative cooling metasurfaces^150–153^. For instance, Sun et al. designed a metasurface featuring a traditional Salisbury screen structure to reflect sunlight and produced the metasurface using transparent and conducting Al-doped ZnO (AZO) with a square geometry to optimize the coupling effect in nanoantennas (Fig. 5d, e)^152^. Broad plasmon resonances were observed in the thermal infrared region, resulting in a 10% increase in the emission efficiency over that of the unstructured AZO (Fig. 5f). Recently, the researchers demonstrated a dynamically tunable thermal emitter based on VO_2_ metasurfaces^153^. By constructing square-shaped microstructures, the metasurface was covered with less VO_2_, leading to a 62% increase in solar transmittance compared to that of the planar emitter. The thermochromic properties of VO_2_ allowed for tunable infrared emissivity, where the strong absorption in hot states came from plasmon effects.

### Photolithography

Photolithography is a method that utilizes light to expose and develop photoresists, followed by the creation of micropatterns on substrate surfaces through an etching process.

Although there are currently limitations to the use of this technique in practical energy-efficient walls and roofs, we believe that the related works could provide inspiration for future developments. Conventional photolithography relies on masks to form patterns, which is effective in producing various arrays^154–157^. Representatively, Heo et al. designed a Janus thermal emitter with different emission characteristics on each side, in which the top and bottom sides exhibited selective and broadband emissions, respectively^155^. The selective side was achieved by photolithographing the quartz substrate into periodic microsquare grooves to induce spoof surface plasmon polariton resonance. Maskless photolithography has also been reported for radiative cooling applications. Lee et al. developed a template with ridge-like periodic nanogratings using laser interference lithography to duplicate Archaeoprepona demophon wing scale structures^158^. By incorporating hierarchical porosities into structured polyvinylidene fluoride (PVDF) cohexafluoropropylene (HFP), the scholars successfully demonstrated enhanced radiative cooling performance with structural colors.

Furthermore, photolithography has been utilized to explore smart materials with switchable cooling performance. Tang et al. fabricated a temperature-adaptive radiative cooler by embedding W_x_V_1-x_O_2_ microblock arrays into barium fluoride (BaF_2_) dielectric layers on a Ag substrate (Fig. 5g)^156^. The resulting 1/4-wavelength cavity serving as a Fabry−Perot resonator amplified infrared absorption in the atmospheric window in the hot state while exhibiting high transparency and minimal infrared absorption in the cold state. The radiative cooling performance was intelligently regulated by temperature (Fig. 5h), which is considered passively smart.

### Nanoimprint lithography

NIL involves the replication of patterned molds on substrates through direct physical contact. This method is superior to other nanolithography techniques in terms of scalability, but its production is limited to 2D and 2.5D structures. Paik et al. used NIL to pattern polymer resists into inverted pillar structures^159^. Following the casting of colloidal VO_x_, lift-off in acetone, and rapid thermal annealing, subwavelength VO_2_ nanopillar arrays were successfully prepared. In addition, the pillars could be designed as multilayer structures containing different W doping contents for a tunable plasmon dipolar response. Liu et al. fabricated Ag-embedded transparent electrodes with high-resolution honeycomb or square patterns^160^. The scholars used a two-step nanoimprinting process to transfer the direct-writing microgroove structures into a soft mold. Then, the mesh electrodes were obtained by filling AgNP ink into microgrooves and sintering. The electrodes were integrated with poly(3,4-ethylenedioxythiophene) (PEDOT):poly(styrenesulfonate) (PSS) to form stable and highly conductive electrodes, which were applied to fabricate polymer dispersed liquid crystal (PDLC) smart windows with a tunable transmittance between 60% and 0.1% in the on and off states, respectively.

## Printing

Printing is a strategy for producing specific shapes by directly depositing colloidal ink on certain areas. Several printing methods, including 3D printing, mech printing, and inkjet printing, have been applied to produce customized structures for solar regulation and radiative cooling. The significant advantages of these printing techniques, especially for applications in building facades, are scalable on the meter-scale and relatively low cost.

### Three-dimensional printing

3D printing is an additive manufacturing technique that is recognized for its advantages of customization, rapid production, and low cost. Notably, 3D printing is an effective approach for manufacturing customized structures.

**Fig. 6:**
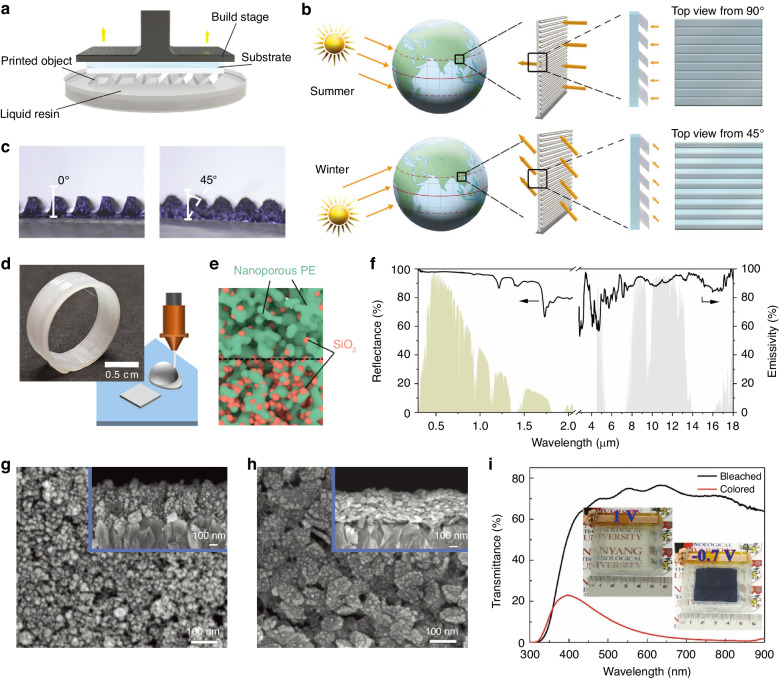
Printing fabrication. **a** Schematic diagram of the fabrication of VO_2_-based composites with tilted microgratings^164^. **b** Schematic diagram of the various exposed surfaces facing direct solar irradiation in summer and winter^164^. **c** Optical microscopy images of the composite films with 0° and 45° tilted microgratings^164^. Reproduced with permission from John Wiley and Sons, copyright 2020. **d** Schematic diagram of the fabrication of diversely shaped polymer composites^168^. **e** Structural diagram of the bilayer composite film with a SiO_2_ concentration gradient^168^. **f** Solar reflectivity and infrared emissivity spectra of the bilayer composite film^168^. Reproduced with permission from the American Chemical Society, copyright 2021. **g** SEM image of the inkjet-printed CeO_2_/TiO_2_ film^177^. **h** SEM image of the inkjet-printed WO_3_/PEDOT:PSS film^177^. **i** Transmittance spectra of the smart window in 300–900 nm at different states with corresponding optical images^177^. Reproduced with permission from John Wiley and Sons, copyright 2017

Various materials have been reported for 3D-printed smart windows, such as thermoresponsive hydrogels^161–163^, thermochromic NP-based polymers^164^, electroresponsive polymer gels^165^, electrochromic films^166^, and magnetic NP-filled polymers^167^. Representatively, Zhou et al. designed micrograting structures on VO_2_-based composites with adjustable tilt angles by digital light processing, as shown in Fig. 6a^164^. This tilted structure could change the exposure surface to sunlight to match the varying solar irradiation in different seasons (Fig. 6b), promoting NIR transmittance contrast. The tilt angle could be further configured to match the different solar elevation angles for cities at different latitudes. Figure 6c shows the printed composite films with tilt angles of 0° and 45°.

This additive manufacturing technique is advantageous for the fabrication of walls and roofs. A range of polymer matrix composites have been developed to fabricate 3D-printed thermal emitters^168–170^. For instance, Zhou et al. presented a nanoporous polymer composite comprising polyethylene (PE) and SiO_2_ for daytime cooling, which could be shaped into diverse structures using 3D printing (Fig. 6d)^168^. The nanopores within PE induced strong scattering, thus enabling reflection to incident sunlight, while SiO_2_ served as a selective emitter, offering high thermal emission in the LWIR region. To minimize the reflection weakening effect in the UV band, the researchers constructed a bilayer film with a distinct SiO_2_:PE weight ratio gradient (Fig. 6e). Consequently, a solar reflectivity of 96.2% in 0.3–2 μm and an infrared emissivity of >90% in 8–13 μm were simultaneously achieved (Fig. 6f). The researchers claimed that 3D printing could extend the practicality of radiative cooling to the construction field.

### Mesh printing

Mesh printing involves using predesigned screens that are formed by fine mesh structures to transfer ink onto objects. During the printing process, materials on the screen openings are transferred to substrates, while materials on the solid mesh are blocked. Therefore, the printed films exhibit periodically gridded patterns at the microscale, which help improve the optical properties of smart windows. Specifically, Lu et al. first reported the performance improvement of VO_2_ thermochromic smart windows via mesh printing^171^. Three sizes (325, 230, and 55 μm) of screen meshes were chosen to print the VO_2_ films. The results showed that the 230- and 55-μm meshes produced gridded VO_2_ with a greater *T*_lum_ than the continuous VO_2_ film. The scholars found that the smaller the mesh size was, the greater the *T*_lum_, regardless of whether the temperature was low or high. The improvement in transparency at short wavelengths could be ascribed to the openings between grids. The performance was expected to be further enhanced by reducing the mesh size. Zhou et al. printed AgNPs onto polyethylene terephthalate (PET) substrates to serve as transparent heaters and integrated them with PNIPAm hydrogels to construct an electrothermochromic window^172^. The high transparency resulted from the high self-alignment of the AgNPs along the mesh wires. The unit openings and the shape characteristics of the mesh were found to be important parameters for controlling the transparency. In addition, the researchers prepared a 10 × 10 cm^2^ sample, highlighting the rapidity and scalability of this mesh printing method. Notably, the affinity among printing materials, meshes, and substrates could play a significant role in determining the final deposited structures.

### Inkjet printing

Inkjet printing, also known as drop-on-demand printing, is a digital printing technique that uses small nozzles or jets to precisely place tiny ink droplets and is widely used in scientific research and industrial production. This process is widely used to fabricate large-scale functional films or create 2D customized patterns.

The advantage of scalable fabrication makes it suitable for smart windows. Large-area VO_2_ thermochromic windows fabricated by this method have been demonstrated^173,174^. These works suggested that increasing the thickness of VO_2_ films by multiple printing could enhance the optical modulation capability. In addition, inkjet-printed large-area electrochromic windows have been reported^175–178^. Cai et al. developed a multifunctional smart window by assembling inkjet-printed ceric oxide (CeO_2_)/TiO_2_ and WO_3_/PEDOT:PSS electrochromic films, where the former acted as the anode and the latter acted as the cathode^177^. Figure 6g, h displays the surface and cross-sectional morphologies of the two films, revealing that the NPs were uniformly distributed. The assembled device with an effective area of 4.5 × 4.5 cm^2^ exhibited high transmittance modulation in 450–900 nm (Fig. 6i). The scholars proved that the performance could be maintained as the device size increased to 18 × 20 cm^2^. The ink surface tension could be controlled by carefully adjusting the solvent component and ratio of inks. The surface tension is considered important for enhancing the affinity between printing materials and substrates and preventing coffee ring effects^179^, which could increase negative haze effects due to their strong scattering. Another important advantage of inkjet printing is the creation of high-resolution patterns. Highly customized patterns could be achieved by inkjet printing, as proven in studies of PDLC^180^ and mechanoresponsive^181^ smart windows.

Inkjet printing is associated with a combination of aesthetics and energy technology for walls. Wang et al. fabricated large-area daytime radiative cooling films that possessed vivid multicolor patterns by printing photoluminescent cesium lead halide quantum dots onto cellulose acetate (CA) NF films^182^. Photoluminescent colorants with different colors were obtained by changing the type and proportion of PbX_2_ (X = Br, I) perovskite quantum dots, which absorbed UV−Vis light in the solar region and emit the desired colors. This work is a valuable attempt to improve the aesthetics and expand the application of quantum dots for radiative cooling.

## Etching

Etching is a technique in surface engineering that removes parts of material surfaces by chemical or physical methods. These methods can be categorized into wet etching and dry etching methods according to the presence of a liquid in the etching process. Although etching directly enables complex 3D structures, both wet and dry etching have the limitations of low scalability and slow processing. The fabrication of functional surfaces for energy-saving windows, walls, and roofs using different wet and dry etching strategies is discussed in this section.

### Wet etching

Wet etching typically relies on chemical reactions between an etchant and selective material surfaces to dissolve or convert the material into soluble byproducts that can be removed, which results in a relatively low cost.

The performance of smart windows can be improved by structural modification with a range of acid and base solutions, including hydrochloric acid (HCl)^183–185^, nitric acid (HNO_3_)^186–188^, hydrofluoric acid (HF)^189^, sulfuric acid (H_2_SO_4_)^190^, oxalic acid (C_2_H_2_O_4_)^191^, and sodium hydroxide (NaOH)^192^. For instance, Bhosale et al. promoted the charge transport of WO_3_/ITO electrochromic films by modifying the surface morphology via HCl etching^184^. The etching process could improve the adhesion of WO_3_ on ITO substrates by changing the ITO structure from crystalline to amorphous. After etching, the annealed WO_3_ films exhibited reduced particle sizes and increased pore sizes, providing open tunnel structures for charge transport during the electrochromic process. As a result, the etched WO_3_/ITO films exhibited relatively good optical modulation (~49% at 630 nm) and stability. In addition, this method could improve the transmission and emission properties of PDMS, which could serve as a static cooling material for windows, as mentioned previously. Gao et al. demonstrated PDMS emitters with random, inverted, and textured pyramids^193^. This structure was manufactured by using silicon templates fabricated through copper-assisted HF etching and subsequent NaOH etching. Compared to flat PDMS, the structured film showed a 2.1% increase in solar transmittance and a 2.7% increase in absorptivity in the atmospheric window.

**Fig. 7:**
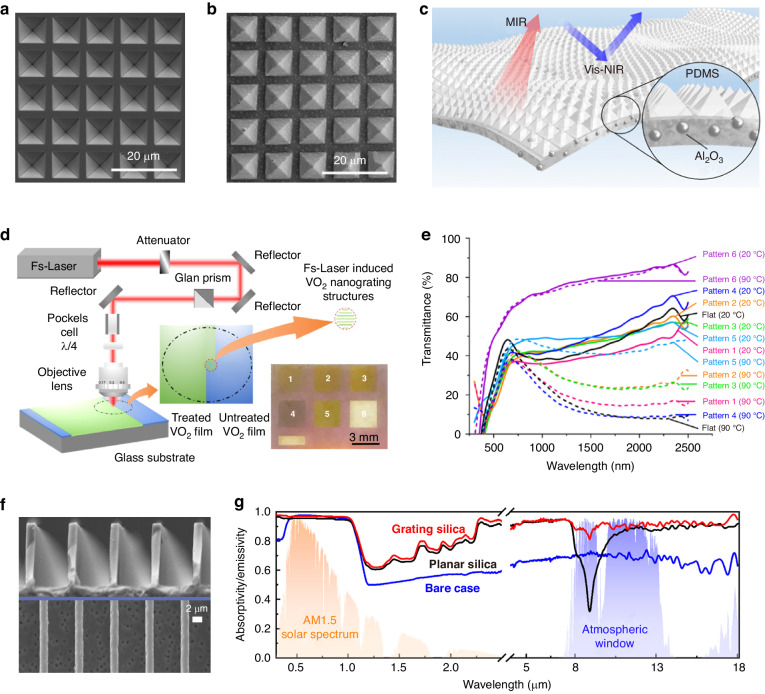
Etching fabrication. **a** SEM image of the template with inverted pyramids^194^. **b** SEM image of the pyramid structures^194^. **c** Structural diagram of bioinspired photonic films^194^. Reproduced with permission from the National Academy of Sciences, copyright 2020. **d** Schematic diagram of the fabrication of femtosecond laser-induced VO_2_^197^. **e** Transmittance spectra of the flat VO_2_ and femtosecond laser-induced VO_2_ films in 300–2500 nm at different states^197^. Reproduced with permission from the American Chemical Society, copyright 2020. **f** SEM images of microgratings on quartz^199^. **g** Emissivity spectra of silica samples with and without microgratings in 0.3–2.5 μm and 5–18 μm^199^. Reproduced with permission from Elsevier, copyright 2022

Wet etching has been utilized to create periodic photonic structures for achieving subambient cooling. Zhang et al. presented a photonic film inspired by longicorn fluff-enabled thermal regulation^194^. The key to the preparation of bionic structures is the formation of inverted pyramid templates (Fig. 7a) by etching grid-masked silicon substrates with potassium hydroxide (KOH). Then, Al_2_O_3_ NP-embedded PDMS films with micropyramid arrays (Fig. 7b) could be obtained by a stamping process performed with these templates through remolding. The micropyramids could provide total internal reflection and gradual refractive index changes, contributing to Vis−NIR reflectivity and LWIR emissivity (Fig. 7c).

### Dry etching

Dry etching is the process of removing material in a vacuum or gas environment using plasma, lasers, and reactive ions. Various studies have been conducted to improve the optical properties of smart windows through dry etching, such as hierarchical Ag grids^195^, textured VO_2_^196^, and nanolattice-patterned VO_2_^197^. Bhupathi et al. fabricated nanolattice-patterned VO_2_ films with controllable periodicity using a femtosecond laser (Fig. 7d)^197^. Compared to the flat VO_2_ film, the structured VO_2_ film simultaneously improved *T*_lum_ and Δ*T*_sol_ (Fig. 7e). The work suggested that further enhanced performance could be expected by using a highly advanced laser system with high numerical aperture objectives and short laser wavelengths. In addition, dry etching could improve the cooling performance of window glass by forming different morphologies on SiO_2_, such as gratings^198,199^, cones^200^, and cylinders^201^. Taking micrograting structures as an example, Zhao et al. used plasma to etch off quartz at a pressure of 4.0 mTorr to produce uniform grating structures with a periodicity of 7 μm and a depth of 10 μm (Fig. 7f)^199^. The SiO_2_ grating was endowed with a high emissivity of 91% in the LWIR region (Fig. 7g).

## Electrospinning

Electrospinning technique, which is one of the most prevalent methods for preparing microfibers (MFs) and NFs, involves the ejection of polymer solutions or molten polymers from a syringe needle by the action of a strong electric field. Multilayered interlaced MF/NF films can be obtained by continuous ejecting and collecting. The production of electrospun fibers is scalable, cost-effective, and highly controllable, which is believed to be beneficial for their application in building facades. Notably, electrospun films are usually endowed with flexible and breathable properties, which is highly desirable for wearable applications. Thus, electrospinning has been promoted as a popular technique for manufacturing cooling textiles for personal heat management^202–206^. In this section, we discuss electrospun films that are potentially applicable to solar management windows and radiative cooling walls and roofs.

**Fig. 8:**
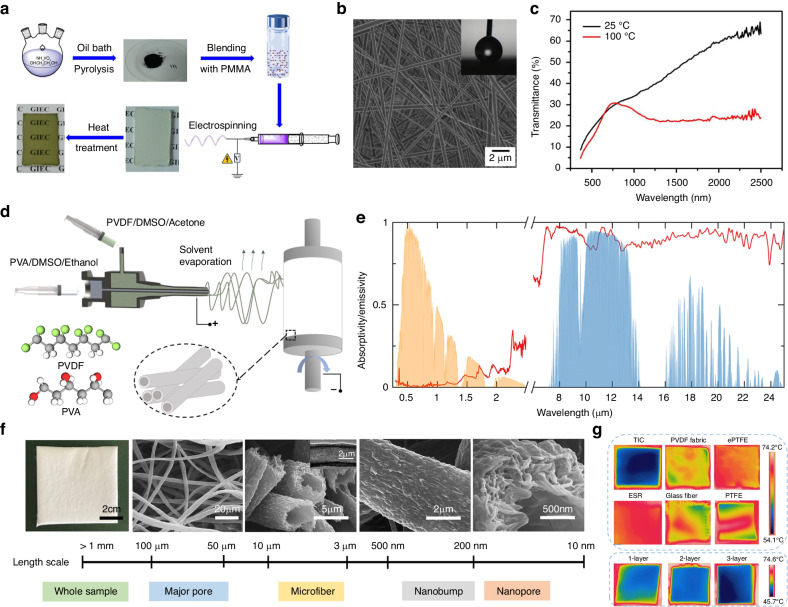
Electrospinning fabrication. **a** Schematic diagram of the fabrication process for the electrospun PMMA–VO_2_ thermochromic films^207^. **b** SEM image of the PMMA–VO_2_ films without heat treatment^207^. **c** Transmittance spectra of the PMMA–VO_2_ thermochromic films in 380–2500 nm at 25 °C and 100 °C^207^. Reproduced with permission from Elsevier, copyright 2017. **d** Schematic diagram of the fabrication process for the hierarchically hollow MFs^217^. **e** Emissivity spectra of the cooling films in the solar and thermal infrared regions^217^. **f** Optical and SEM images of the hierarchically hollow MFs^217^. **g** Infrared images of the hollow fiber-based cooler and contrast samples on a heat source^217^. Reproduced with permission from the American Chemical Society, copyright 2021

In general, most chromogenic materials cannot be directly applied in electrospinning. To address this dilemma, an effective strategy is to employ suitable polymer matrices to modify these materials. For instance, Lu et al. applied electrospinning for the first time in the preparation of VO_2_ thermochromic films using PMMA as the matrix (Fig. 8a)^207^. The SEM image in Fig. 8b displays the PMMA–VO_2_ composite film consisting of cylindrical fibers with uniform diameters. Since the VO_2_ NPs distributed in PMMA were isolated from the air, their antioxidation capacity could be enhanced. After hot treatment, the film exhibited transparent characteristics and thermochromic properties with a Δ*T*_sol_ of 6.88% (Fig. 8c). Through a similar method, the scholars produced thermochromic films by hot pressing single-layer and multilayer PMMA–VO_2_ composites^208^. Based on the electrospinning method, a variety of composite materials have been developed for electrochromic films, including polyvinylpyrrolidone (PVP)−TiO_2_^209,210^, PVP−Cu-doped nickel oxide (NiO)^211^, PEO–WO_3_^212^, poly(vinyl butyral-co-vinyl alcohol-co-vinyl acetate) (PVB–CVA−CVAc)−WO_3_^213^, PMMA–PEDOT–WO_3_^214^, and PVDF–HFP–4-amino-2,2,6,6-tetramethylpiperidine-1-oxyl (4-amino-TEMPO)^215^.

Regarding heat management in walls and roofs, polymers with high emission performance can be used directly as spinning precursors to fabricate cooling films. The formation of hierarchical structures during the electrospinning process is effective in improving the solar scattering efficiency, which is an important parameter in subambient radiative cooling. For example, Kim et al. produced slender polyacrylonitrile (PAN) NFs and investigated the relationship between the fiber morphology and the PAN solution concentration^216^. The researchers reported that the ellipsoidal and cylindrical geometries brought about a strong scattering efficiency to sunlight due to their dielectric resonances. Furthermore, Zhong et al. utilized the coaxial electrospinning method to fabricate hierarchically hollow MFs, as shown in Fig. 8d^217^. The cooling films formed by these fibers offered not only a high solar reflectivity of 94% but also a high LWIR emissivity of 94% (Fig. 8e). Together, MFs, nanobumps, hollow structures, and nanopores (Fig. 8f) contributed to efficient solar scattering and effective thermal insulation. When subjected to a heat source, the cooling films exhibited thickness-dependent thermal insulating performance (Fig. 8g). Additionally, electrospun fibers with enhanced solar scattering, such as CA^218^, PVDF−Al_2_O_3_^219^, poly(vinyl alcohol) (PVA)−SiO_2_^220^, and PVDF−tetraethoxysilane (TEOS)^221^, have been widely used in wall and roof cooling. The processing efficiency is an important factor that should be considered in the practical mass production of large-area cooling films.

## Conclusions and outlook

In this review, we summarize the recent progress in advanced micro/nanofabrication techniques for heat management materials for potential energy-efficient building facades, including windows, walls, and roofs. The fabrication methods include coating, vapor deposition, nanolithography, printing, etching, and electrospinning. We also propose our perspectives on the advantages and disadvantages of these fabrication methods, as listed in Table 1. Materials and fabrication developments are intricately connected. Rapid advancements in materials drive corresponding needs for associated fabrication methods. Moreover, innovative fabrication techniques enhance material performance, revealing materials with novel properties. For example, the colloidal lithography method for VO_2_ produces a material with precisely controllable nanoscale structures, leading to the systematic investigation of localized surface plasmon resonance^63^.

**Table 1:** Advantages and disadvantages of typical micro/nanofabrication techniques

Fabrication methods		Pros	Cons	Remarks
Coating	Spray coating	• Meter-scale and rapid production • Low cost • Relatively high quality control on centimeter scale	• Unsuitable for complex 3D structures • Low quality control on submicroscale	/
	Dip coating			• Low equipment requirements
	Spin coating			• Limited to subinch-scale production
	Roll-to-roll processing			• Cost will be high if integrated techniques are expensive
Vapor deposition	CVD	• Inch-scale production • Suitable for complex 3D structures • High quality control down to nanoscale	• Relatively slow production • Relatively high cost • High equipment requirements	/
	ALD			• High cost • Ultrahigh quality control down to atomic scale
	PVD			/
Nanolithography	Colloidal lithography	• Inch-scale production • Suitable for complex 3D structures • High quality control down to nanoscale	• Slow production • Limited to specific 2D and 2.5D structures formed by colloidal masks	• Meter-scale production • Relatively low cost • Low equipment requirements
	EBL		• Slow production • High cost • High equipment requirements	• Limited to subinch-scale production • Ultrahigh quality control down to atomic scale
	Photolithography			/
	NIL			• Meter-scale production • Limited to 2D and 2.5D structures
Printing	3D printing	• Meter-scale and relatively rapid production • Relatively low cost • Suitable for 2D-to-3D structures • High quality control down to microscale	• Relatively low quality control on nanoscale • High equipment requirements	/
	Mesh printing			• Limited patterns depending on screen meshes
	Inkjet printing			/
Etching	Wet etching	• Inch-scale production • Suitable for complex 3D structures • High quality control down to microscale	• Slow production	• Low cost • Relatively low equipment requirements
	Dry etching			/
Electrospinning		• Meter-scale production • Low cost • High quality control down to nanoscale	• Slow production • Unsuitable for complex 3D structures • High equipment requirements	• Suitable for hierarchically structured fiber networks

An ideal fabrication method is expected to have high throughput, high precision, and the capability to create complex 3D structures. However, meeting these features simultaneously is extremely challenging, and there always seems to be a trade-off among them. For example, 2D/3D printing methods are generally recognized as high-throughput methods compared to nanolithography techniques, but their precision is much lower than that of nanolithography. Recent developments in nanoscale 3D printing based on two-photon polymerization have achieved high precision down to sub-100-nm resolution^222,223^, but the fabrication process is much more time-consuming than other single-photon 3D printing methods, such as digital light processing. This process makes such nanoscale 3D printing more applicable for micro/nano-optical devices (such as microlenses) but not suitable for meter-optical devices (such as smart windows and radiative cooling walls and roofs). NIL is promising for achieving both high throughput and high precision, but this method is limited to producing 2D and 2.5D structures. Future development is expected to improve the throughput, precision, and the capability to create complex 3D structures.

In addition, most materials for dynamic solar or thermal emission management require at least a meter scale to achieve adequate power and economic benefits in practical applications, which is very different from other micro/nanophotonic devices that are characterized by their unique functionality and can profit from small products. Regarding energy-saving materials for windows, walls, and roofs in the construction industry, both mass production and relatively low cost are essential prerequisites. In practice, the best methods are direct spray coating and brush painting, which are similar and relatively simple processes to dip coating. These methods are facile, relatively low cost, and applicable for existing walls and roofs. The methods that exhibit relatively high equipment requirements, such as roll-to-roll processing, vapor deposition, 3D printing, dry etching, and electrospinning, are more suitable for new production in factories followed by installation in buildings. These products include glass, window foils, and wallpapers. Chen et al. demonstrated a good example of producing VO_2_-based energy-saving glasses in several square meters through a roll-coating method^224^. The mass production and low cost that are demanded in the practical field temporarily eliminate the possibility of using fabrication methods of ALD and EBL, which are generally considered to be too expensive and time-consuming for materials in the building industry. However, we believe that these techniques are good for demonstrating the material−property relationship at the laboratory level, and a switch to a high-throughput method for mass production should be implemented before practical application. Potential solutions to this issue can be to modify material design to adapt to cost-effective fabrication methods, and a rational combination of two or more fabrication techniques may be helpful, especially those based on composite materials. Moreover, during mass production processes, the environmental impacts should be considered. The product materials themselves, waste materials, byproducts, and recycling processes should all be eco-friendly.

Beyond fabrication methods, this development holds great promise for energy conservation and carbon emissions reduction in the building sector, which accounts for ~40% of the energy in developed regions, such as the United States and the European Union^4,144^. Energy-saving smart windows, walls, and roofs have great potential in the field. The solar and infrared radiation powers typically range from 800 to 1200 kW/m^2^ and 50 to 120 kW/m^2^, respectively, in most regions. Taking Singapore as an example, the annual solar and infrared radiation are ~1600 and ~440 kWh/m^2^, respectively (considering an infrared radiation power of ~50 kW/m^2^ in humid air)^225,226^. The utilization of a 1 m^2^ roof accounts for a total power of ~2040 kWh annually, equivalent to ~450 dollars in electricity costs and ~800 kg of carbon emissions locally. Therefore, even a 1% improvement in the performance of solar and infrared thermal emission from building facades is considerable, given the extensive surface area of buildings.

By summarizing the progress, we hope this review will facilitate the development of advanced micro/nanofabrication methods for modifying the spectral properties of energy-efficient building facade materials.
